# The Association of Women's Participation in Farmer-Based Organizations with Female and Male Empowerment and its Implication for Nutrition-Sensitive Agriculture Interventions in Rural Ghana

**DOI:** 10.1093/cdn/nzac121

**Published:** 2022-07-25

**Authors:** Aishat Abdu, Grace S Marquis, Esi K Colecraft, Naa D Dodoo, Franque Grimard

**Affiliations:** School of Human Nutrition, McGill University, Montreal, Quebec, Canada; School of Human Nutrition, McGill University, Montreal, Quebec, Canada; Department of Nutrition and Food Science, University of Ghana, Legon, Ghana; Regional Institute for Population Studies, University of Ghana, Legon, Ghana; Department of Economics, McGill University, Montreal, Quebec, Canada

**Keywords:** nutrition-sensitive agriculture, empowerment, nutritional status, food security, gender, agriculture, farmer-based organizations, rural, Ghana

## Abstract

**Background:**

Few studies have examined the influence of women's participation in farmer groups on female and male empowerment, which is considered essential to improving nutrition.

**Objectives:**

The study aimed to *1*) assess the empowerment of Ghanaian women farmers, 1 adult male family decision-maker per household, and the household gender equality; and *2*) investigate the relation of empowerment and household gender equality with women's participation in farmer-based organizations (FBOs), women's and men's nutritional status, and household food security.

**Methods:**

A cross-sectional study investigated secondary outcomes using baseline data from a nutrition-sensitive agriculture intervention implemented through FBOs in rural Ghana. Existing FBOs in 8 communities were selected based on 6 criteria (e.g., participation level, readiness to change). Female FBO (*n *= 166) and non-FBO (*n *= 164) members together with a male family member (*n *= 205) provided data on individual and household characteristics; empowerment was measured across 11 indicators with the project-level Women's Empowerment in Agriculture Index. Generalized linear mixed models tested the associations of empowerment and household gender equality with FBO membership, nutritional status, and household food security.

**Results:**

Women's FBO membership was associated with an increased likelihood of women's empowerment [adjusted odds ratio (aOR): 3.25; 95% CI: 1.97, 5.33] and household gender parity (aOR: 2.82; 95% CI: 1.39, 5.84) but not men's empowerment. Household food insecurity, but not nutritional status, was positively associated with women's FBO participation and individual empowerment indicators (financial services). Food insecurity was negatively associated with the women's empowerment indicator related to attitudes about domestic violence [adjusted β coefficient (aβ): −0.78; 95% CI: −1.35, −0.21] and men's overall empowerment (aβ: −0.79; 95% CI: −1.58, −0.01).

**Conclusions:**

Understanding the complexity in which FBO participation, empowerment, nutritional status, and food security are linked is critical in designing interventions that promote gender equality and improved nutrition.

This trial was registered at clinicaltrials.gov as NCT03869853.

## Teaser

Women's participation in farmer-based organizations and household food security are differentially linked to women's and men's empowerment.

## Introduction

In Ghana persistent gaps exist between women and men across the country, particularly among rural populations engaged in agriculture (1, 2). Nearly 50% of rural Ghanaian women are employed as farmers, yet they lag behind men in accessing agricultural resources such as productive assets, inputs, labor, and extension services (2, 3). Estimates show Ghanaian men own 3 times more farms, have larger landholdings, and are more likely than women to access formal financial services in the rural areas (3, 4). Women are more likely to be employed as unpaid family workers and face time constraints due to time allocated to domestic activities (89% of women spending ≥10 h/wk compared with 65% of men spending between 0 and 10 h/wk on these), further affecting women's productivity in the agriculture sector (3, 5). Closing the gender gap has been advocated as a human right and a key step to achieving the Sustainable Developmental Goals related to improved nutritional status and household food security (6–8).

Empowerment is the process by which people expand their capabilities to make choices that are important to them and is key to reducing the gender gap (9). Community groups have been shown to be effective in providing knowledge and resources needed for individuals to exercise their choices at the individual, household, and community levels (9, 10). Participation in farmer-based organizations (FBOs) is one pathway that may contribute to the empowerment of rural women (10, 11). A recent study showed women's membership in dairy producer organizations improved their use of income, ownership and decision-making over land and assets, and control over productive decisions (12). Women's empowerment in agriculture has been linked with nutrition through 3 theoretical pathways (13). Yet, evidence from studies looking at this linkage are limited owing to methodological limitations and contextual differences in definitions of empowerment (13–15). However, researchers have demonstrated the benefits of improvement in different domains of empowerment, including *1*) women with control over resources invested in the nutrition of the household, *2*) increased group membership and ownership over assets positively correlated with household food security, and *3*) more decisions related to agricultural production was negatively associated with the risk of obesity among women (16–20). Nevertheless, few studies have investigated the relation between empowerment in agriculture and women's own nutritional status (13). Available evidence on the empowerment indicators also suggests differential linkages across regions, which may influence the design of local policies and interventions to empower women (7, 15).

Most studies have assessed the impact of group participation on women's empowerment only (21). Few researchers have looked at the impact of women's participation in groups on the empowerment of other household members (22–24). Although studies have reported on improvement in household income and diet quality, some evidence has pointed toward changes in intrahousehold dynamics when women's status and bargaining power were improved, including male disempowerment and feelings of threats to male authority (25–27). In other cases, male partners reported reducing their contribution to household food expenses (26). Changes in household dynamics could negatively affect some domains of women's empowerment, increasing intrahousehold conflicts to the detriment of the nutritional status of household members and household food insecurity (28, 29).

FBOs are promoted in Ghana for agriculture and rural development (30). In the 4743 registered FBOs, ∼42% of the members were women in 2018 (31). The Government of Ghana views delivering extension services to groups via local institutions as an efficient and cost-effective way of reaching farmers, and this has been promoted through policies such as the Food and Agriculture Sector Development Policy (FASDEP II) (30, 32). As such, most farmers join FBOs voluntarily to access technical support from agriculture extension agents (AEAs) and benefit from governmental and nongovernmental organization (NGO) projects that provide loans, input, and training support to groups rather than individuals (33). They also join groups to benefit from labor exchange, pooling of resources, and accessing credit through local credit schemes or formal institutions. About 58% of FBOs in Ghana were reported in 2010 as externally started (i.e., started by government institutions or NGOs), whereas the remainder were started by individuals living within the same communities to access support from the government and NGOs. On average, FBOs comprise 36 members and meet regularly to access support. These groups also carry out a range of activities which have been used to categorize them into production, processing, marketing, and multipurpose FBOs. Although FBOs are a platform for women farmers to access resources and services that allow them to expand their choices, gaps remain in gender mainstreaming in the Ghana agriculture sector (3). Women's participation in these groups has been rarely assessed through a gender lens to monitor progress that will inform policy decisions (2).

Given the highlighted gaps in the literature and the interest in empowerment as a pathway to gender equality and improved nutrition-related outcomes, this study aimed to *1*) assess the empowerment of Ghanaian women farmers who are members and nonmembers of FBOs, 1 adult male decision maker per household, and the household gender equality; and *2*) investigate the relations of women's participation in FBOs, adult nutritional status, and household food security with women's and men's empowerment and household gender equality.

## Methods

This cross-sectional study investigated secondary outcomes using baseline data collected as part of Scaling Up Women's Agripreneurship through Public-Private Linkages to Improve Rural Women's Income, Nutrition, and the Effectiveness of Institutions in Rural Ghana (LINkINg Up; NCT03869853), a quasi-experimental, nutrition-sensitive agriculture intervention designed to improve the quality of life of rural Ghanaian women agricultural entrepreneurs and their families in 3 districts of the Eastern Region of Ghana. The LINkINg Up initiative was designed to sustainably build on lessons learned from a previous cluster-randomized controlled trial, Nutrition Links (NL), an integrated agriculture and nutrition education intervention implemented in the Upper Manya Krobo District (UMKD) of Ghana (2014–2017), by scaling up activities and services to women and their households (34). The rationale was that sustained integrated approaches that increase agricultural productivity, diversify incomes, and enhance knowledge and skills among all stakeholders are needed to improve the well-being of rural communities. As a result, the LINkINg Up coordinators engaged with the NL sustainability committee that was formed during the project to guide in the planning and selection of districts for the scale-up of activities and services. The LINkINg Up initiative partnered with local institutions [Department of Food and Agriculture, Upper Manya Krobo Rural Bank, District Assembly (the local government), Ghana Health Service, and Ghana Education Service in the UMKD, Lower Manya Krobo District (LMKD), and Yilo Krobo District (YLKD)] that were selected through stakeholder engagement, to provide loans (i.e., poultry input loan package or vegetable loan input package) and agriculture and nutrition education to female members of existing FBOs. The initiative adopted Heifer's Passing on the Gift® community development approach where the repayment of loans provided funds for a new set of participants following a 12-mo repayment cycle. As such, the study sample was divided into 2 groups, phase 1 (2019–2020) and phase 2 (2021–2022), with each phase accounting for 50% of the women recruited as study participants from the selected FBO groups. Whereas loan inputs were only provided to women during their corresponding phase cycle period, capacity building, technical and financial trainings, as well as other services provided by the partnered institutions were open to all women in the FBO groups (both phase 1 and phase 2 parti-cipants). Note that the LINkINg Up activities were still ongoing for the phase 2 participants at the time of writing this article.

### Sample

A list of all existing FBOs with ≥40% female membership for the 3 districts in the Eastern Region of Ghana were collected from a database of registered FBOs available at the Ministry of Food and Agriculture. The 2017–2018 regional report on FBOs in Ghana estimated that ∼2475 (27.3%) out of 9072 members were women in the 496 FBOs in the Eastern region (31). Active FBOs in the selected project districts (UMKD, *n *= 7; LMKD, *n *= 7; YLKD, *n *= 10) were shortlisted to be assessed against the inclusion and exclusion criteria defined by the LINkINg Up partners to determine participation in the initiative. The FBO executive officers (i.e., leaders, secretary) and local AEAs gathered information for each of the FBOs for the previous 4 mo on *1*) percentage of female members; *2*) meeting schedule (i.e., frequency, expected meetings, and actual meetings held) and location; *3*) number of meetings, dates, and average attendance; *4*) previous work with NGOs; and *5*) FBO activities (e.g., production, processing). Using this information, the AEAs evaluated the FBOs using a rating scale of 1–5 (5 = highest) on the members’ level of participation, leadership potential, congeniality within the group, ease of collaboration, group's need and potential impact, and readiness to change. A final score [UMKD: 29.3 ± 3.5 (mean ± SD); LMKD: 19.0 ± 0.7; YLKD: 17.8 ± 2.0] was generated for each FBO by adding the ratings in each of the aforementioned categories. The highest-ranked FBOs by the AEAs were then evaluated for distance, proximity to each other, and alignment with the economic activities proposed by the LINkINg Up initiative, and a total of 8 FBOs (UMKD, *n *= 2; LMKD, *n *= 1; YLKD, *n *= 5) were chosen to participate. The focus on active FBOs (i.e., those that were meeting regularly and carrying out activities together) was to test the feasibility and sustainability of the LINkINg Up initiative for scaling up among other existing FBOs in the districts.

The selected FBOs had on average 34.4 members, were women-only (*n *= 5) and mixed (*n *= 3) groups, and engaged in different activities which included production, processing, labor support, and village savings and loan groups. One of the FBOs was a multipurpose group, 4 were solely a production group, and the remainder were a combination of production, processing, and either savings/loans or social support groups. Most (*n *= 5) of the FBOs were formed by an AEA and the others were self-formed then registered with the support of the AEA at the Department of Food and Agriculture. All members reported joining the groups voluntarily. The selected FBOs reported that group meetings were held either weekly or twice monthly. In most cases, the FBO members were from the same communities.

In most of the selected FBOs, all women were enrolled to participate in the project. The few women who chose not to participate did not differ in demographic characteristics from the women who partici-pated in the project. Because the project was divided into 2 phases, larger FBOs self-selected the members who would participate in the first and second phases of the project. We checked for differences between these participants and only found a significant difference in marital status (*P *= 0.043) between the phase 1 and 2 female participants and no difference in other characteristics (such as age, or education for women and men). For all the households where a female FBO member enrolled to participate, a male adult living in the house and who identified as the primary male decision maker within the same household was also recruited for the project surveys. The rationale for including men was to assess women's empowerment relative to the male adult decision maker in the household. Some of these males (UMKD, *n *= 29; LMKD, *n *= 47) were also members of the FBO groups that were mixed but were not selected based on this characteristic. For households where both the woman and man were in the FBOs, they were both registered. However, in this study we only focus on the woman's FBO membership. Hence, all the results on the relation between FBO participation and outcomes of interest are referring to the woman's participation in FBOs, so we interpret this study's results as the benefits related to the woman's participation.

In addition to the FBO members, a sample of women who were not members within each FBO community were recruited as a comparison group for the project. These participants were selected randomly from a census of farmers who were not members of FBOs within the same communities. In 1 district, enumerators faced challenges finding the randomly selected residents because they were not home and replaced them at random with the next available person (e.g., neighbor). Similarly, male adults in the comparison group households were also recruited into the project.

The LINkINg Up project recruited 330 households with 166 women (82 in phase 1 and 84 in phase 2) who were FBO members and 164 women (83 in phase 1 and 81 in phase 2) who were nonmembers. In addition, the staff enrolled 205 adult male family members (201 spouses or partners, 1 father, and 3 sons) who self-identified as primary decision makers within the household. Although the project aimed to recruit men from all 330 households, this was not possible with our sample because 25.4% (*n *= 84) of our households were female-headed and the remainder of men in the identified dual-adult households (*n *= 41) were not available (i.e., owing to illness, travel) to be interviewed during the period of data collection. The comparison of women in female-headed households with those in dual-adult households showed significant differences in age, ethnicity, marital status, and household size. Whereas, the comparison of the characteristics of women in the 205 households where a man was interviewed with those in the households (*n *= 41) where a man was not interviewed showed only a significant difference in marital status. As a whole, the comparison of women paired with men and those women not paired with men showed significant differences in ethnicity, marital status, age, household size, and headship.

### Data collection

The data for this analysis were collected by trained field staff using electronic tablets between November 2019 and January 2020 for phase 1 and between November 2020 and January 2021 for phase 2. The primary outcomes of the study include empowerment (women's empowerment, male empowerment, and household gender parity), women's and men's BMI (in kg/m^2^), and household food security.

Empowerment outcomes were measured using the project-level Women's Empowerment in Agriculture Index (pro-WEAI), a standardized tool to capture the empowerment and agency of women and men in the agriculture sector as well as the gender gap within the household (35). This survey was administered to both the recruited woman and man in each household. For households that did not have a man enrolled in the project, only the woman was assessed. Empowerment was measured across 11 equally weighted indicators: *1*) autonomy in income, *2*) self-efficacy, *3*) attitudes about domestic violence, *4*) control over the use of income, *5*) input in productive decisions (participation in decisions for household agriculture activities), *6*) asset ownership (land and household assets), *7*) mobility, *8*) access to and decisions on financial services, *9*) work balance, *10*) group membership, and *11*) membership in influential groups. Information for 1 pro-WEAI indicator, respect among household members, was incomplete for female-only households and therefore was not used in this study. The survey questions on group types for calculating the empowerment indicators related to group membership and membership in influential groups did not include FBOs as 1 of the response options.

Weight (kg) and height (cm) were measured in duplicate using standardized methods with a digital scale (Tanita Corporation of America, Inc.) and stadiometer (Shorr Production), respectively. Household food security was measured using the 15-item Latin American and Caribbean Food Security Scale (36). Data were also collected on the following covariates: sociodemographic characteristics (age, education, marital status, ethnicity) and household characteristics (family composition, and assets).

### Data analysis

Empowerment was assessed in 3 ways: overall empowerment (women and men), empowerment in the individual indicators, and household gender parity. The empowerment variables were calculated as follows. First, women and men were independently classified for each of the 11 indicators (adequate = 1; inadequate = 0) based on their survey responses compared to the pro-WEAI predetermined thresholds defined in Malapit et al. (35). Second, the empowerment score for each participant was calculated by multiplying the binary variable (0 or 1) for each indicator by the weight of 0.09 (all indicators weighted 1/11) and summing up the scores. Third, participants were classified as empowered (score ≥ 0.80) or disempowered (score < 0.80). We chose achieving empowerment on between 8 and 9 indicators (cutoff ≥ 0.80) as our cutoff because 8 out of 11 indicators (cutoff ≥ 0.72) was lower than what was recommended and 9 out of 11 indicators (cutoff ≥ 0.82) was greater than what was recommended. The analysis with the individual indicators focused on the 5 indicators (attitudes about domestic violence, mobility, access to and decisions on financial services, group membership, and membership in influential groups) that were significantly different between women FBO and non-FBO members.

The gender parity variable was constructed only for the households (*n *= 205) where a woman and a male pair were interviewed (35). An intrahousehold empowerment gap was determined by comparing the empowerment scores of each woman and her male pair. All households where a woman was empowered irrespective of the male adult's score, or where she was not empowered but her score was equal to or greater than her male pair's score, were classified as achieving gender parity. Households where a woman was not empowered and her score was lower than the male pair's score were classified as households lacking gender parity.

The BMI was calculated as weight (kg)/height (m^2^) and used as a continuous variable. For household food security, households were categorized based on the number of affirmative answers; this differed for households without children [food secure (0), mildly food insecure (1–3), moderately food insecure (4–6), and severely food insecure (7–8)] and households with children [food secure (0), mildly food insecure (1–5), moderately food insecure (6–10), and severely food insecure (11–15)]. Finally, a binary variable was created: food secure, food insecure (including mildly, moderately, and severely).

Household size was included as a continuous variable. All other explanatory variables were categoric: FBO membership (member, nonmember), education (none, primary, secondary, or higher), age (<35, 35–44, 45–54, ≥55 y), marital status (married/cohabiting, not married/cohabiting), ethnicity (Krobo, other), and project phase (phase 1, phase 2).

The wealth variable was derived from a principal component analysis of 18 household assets (improved water source, floor materials, wall materials, roof materials, toilet facility, cooking fuel, ownership of agricultural land, small livestock, nonmechanized farm equipment, mechanized farm equipment, owns house or building, electricity, motorcycle, bicycle, cellphone, radio, television, and refrigerator). Wealth scores were extracted from the first component and categorized by tertile (low, middle, high).

Descriptive statistics based on women's FBO participation for women and their male family member were tested using independent Student's *t* test for continuous variables and a chi-square test of independence for categoric variables. Continous variables were presented as mean ± SD.

#### Primary analysis

To develop the final adjusted models, chi-square tests, Student's *t* test, and unadjusted logistic or linear regressions were used to examine bivariate associations between the outcome variables (empowerment, BMI, and food security) and explanatory variables. The independent variables with a *P* value < 0.10 in the bivariate analysis were included into the final models to control for covariates. Relevant variables that were associated with the outcomes in published literature were also included in the final models even if they were not significant in the bivariate analysis. We also included project phase in all our models. Multicollinearity between explanatory variables was checked by the variance inflation factor; no model had a value >10.

The association between women's FBO membership and the empowerment variables was tested with a generalized linear mixed model (PROC GLIMMIX) adjusting for covariates and the random effects of clusters (i.e., community). The random effect of cluster was not statistically significant in our models but was still retained in the analysis. The association of women's FBO membership and the empowerment variables with nutrition outcomes (women's BMI, men's BMI, and household food security) was initially tested with a generalized linear mixed model (PROC GLIMMIX) that included *1*) the interaction between empowerment and FBO membership, *2*) covariates, and *3*) the random effects of clusters. The interaction term was not significant in any of our models and the results did not vary with or without the interaction term. Therefore, the interaction term was dropped from the models. We adjusted the α levels and corrected the CIs for all covariates with >2 categories using Dunnett's method (37). We performed ex post power analysis of minimum detectable differences for each of our models with empowerment outcomes (38). All analyses were conducted using SAS version 9.4 (SAS Institute Inc.). The level of significance was set at <0.05.

#### Secondary analysis

Two types of analysis were conducted for women and men in separate mixed-effects models. First, the association between women's FBO membership and each individual empowerment indicator as an outcome was tested with a generalized linear mixed model (PROC GLIMMIX) adjusting for covariates and the random effects of clusters. The *P* values for the 5 individual empowerment indicators significantly different between women FBO and non-FBO members were corrected for multiple hypothesis testing following the Benjamini et al. (39) method for *q* value corrections (40). Second, the association of the 5 individual indicators as covariates with nutrition outcomes (women's BMI, men's BMI, and household food security) was tested with a generalized linear mixed model (PROC GLIMMIX) adjusting for other covariates and the random effects of clusters.

### Ethical approval

The ethical approval for this study was obtained from the institutional review boards of McGill University (# 377-0219) and the University of Ghana College of Basic and Applied Sciences (#035/18-19). All partici-pants provided informed written consent after project staff provided a detailed explanation of the project as well as an understanding that their anonymized data may be used in future analyses. Data were registered and stored in a secured server and the permission to access data was granted by the principal investigators (GSM, EKC) with personal identifiers removed. Participants received nonmonetary compensation (i.e., bar of soap, a small farm implement) for the completion of the surveys. Participants were made aware that there were no immediate benefits but their participation in the research activities would help guide the development of interventions to enhance the work and well-being of women engaged in agriculture-based livelihood activities.

## Results

### Demographic characteristics

This analysis included 316 households (316 women and 198 men); 14 women and 7 men had incomplete data. There were 191 households with no missing data for both the woman and man. The proportion of female adult households did not differ between FBO members and non-FBO members (24.2% compared with 27.7%; *P* = 0.48). Over half of the households reported experiencing food insecurity with a higher proportion reported by FBO households (Table 1). There were differences in household characteristics by phase, with a higher rate of food insecurity in phase 1 than in phase 2 (65.5% compared with 52.2%; *P* < 0.02). There were no differences in individual characteristics of male pairs of the FBO and non-FBO members. Women FBO members had a higher mean BMI than non-FBO members (Table 1).

**TABLE 1:** Characteristics and empowerment indicators of women and men farmers in rural Ghana, by woman's FBO membership^1^

	Women			Men		
Variables	FBO (*n* = 157)	Non-FBO (*n* = 159)	*P* value^2^	FBO (*n* = 101)	Non-FBO (*n* = 97)	*P* value^2^
Individual						
Age group, y			0.34			0.39
<35	32 (20.4)	44 (27.7)		11 (10.9)	17 (17.5)	
35–44	44 (28.0)	38 (23.9)		25 (24.8)	28 (28.9)	
45–54	43 (27.4)	35 (22.0)		29 (28.7)	25 (25.8)	
≥55	38 (24.2)	42 (26.4)		36 (35.6)	27 (27.8)	
Ethnicity^3^			0.95			0.46
Krobo	128 (81.5)	130 (81.8)		86 (85.2)	86 (88.7)	
Education^4^			0.06			0.92
None	43 (27.4)	53 (33.3)		9 (8.9)	10 (10.3)	
Primary	62 (39.5)	43 (27.1)		24 (23.8)	24 (24.7)	
Secondary or higher	52 (33.1)	63 (39.6)		68 (67.3)	63 (65.0)	
Marital status^5^			0.94			0.16
Married/cohabiting	117 (74.5)	119 (74.8)		99 (98.0)	97 (100)	
BMI, kg/m^2^	26.1 ± 6.5	24.7 ± 5.9	0.04	23.1 ± 6.9	22.8 ± 10.6	0.81
Household						
Size, *n*	5.1 ± 1.9	5.2 ± 2.0	0.46	5.5 ± 2.4	5.2 ± 1.6	0.23
Wealth^6^			0.30			0.44
Low	49 (31.2)	58 (36.5)		29 (28.7)	33 (34.0)	
Medium	49 (31.2)	54 (33.9)		31 (30.7)	33 (34.0)	
High	59 (37.6)	47 (29.6)		41 (40.6)	31 (31.9)	
Food security^7^			0.05			<0.01
Food insecure	100 (63.7)	84 (52.8)		68 (67.3)	46 (47.4)	
Phase of enrollment						
Phase 1	74 (47.1)	80 (50.3)		63 (62.4)	49 (50.5)	
Phase 2	83 (52.9)	79 (49.7)		38 (37.6)	48 (49.5)	
Empowerment^8^						
Empowered (1 = empowered)^9^	109 (69.4)	66 (41.5)	<0.001	73 (72.3)	61 (62.9)	0.15
Household gender parity^10^	73 (76.0)	56 (58.9)	0.01			
Empowered in individual indicators^11^						
Attitudes about domestic violence, yes	119 (75.8)	103 (64.8)	0.03	89 (88.1)	80 (82.5)	0.26
Access to and decisions on credit, yes	108 (68.8)	89 (55.9)	0.01	75 (74.3)	61 (62.9)	0.08
Mobility, yes	121 (77.1)	99 (62.3)	0.004	70 (69.3)	66 (68.0)	0.84
Group membership, yes	141 (89.8)	121 (76.1)	0.001	79 (78.2)	64 (65.9)	0.05
Membership in influential groups, yes	116 (73.9)	82 (51.6)	<0.001	66 (65.4)	52 (53.6)	0.09

1 Values are *n* (%) or mean ± SD. FBO in the women's and men's columns indicates that the respondent woman in the household was participating in an FBO; non-FBO in the women's and men's columns indicates that the woman of the household was not participating in an FBO. FBO, farmer-based organization; pro-WEAI, project-level Women's Empowerment in Agriculture Index.

2 Independent Student *t* test for continuous variables; chi-square test of independence for categoric variables.

3 Krobo, the local ethnic group, was compared with others (Akan, Ewe, Ga, among others).

4 Highest level of education completed.

5 Married/cohabiting compared with not married or cohabiting.

6 Wealth was categorized by tertile for the first component of a principal components analysis of 18 household assets [improved water source, floor materials, wall materials, roof materials, toilet facility, cooking fuel, ownership of agricultural land, small livestock, nonmechanized farm equipment (i.e., hand tools), mechanized farm equipment (i.e., tractor), house or building, electricity, motorcycle, bicycle, cellphone, radio, television, and refrigerator].

7 Food security was classified based on the 15-item Food Insecurity Experience Scale (36), as food secure and food insecure (which included mildly, moderately, and severely food insecure).

8 Empowerment outcomes were measured using the pro-WEAI (35).

9 Empowered: scored ≥80% in the 11 empowerment indicators (≥0.80).

10 Household gender parity was calculated only for the households (*n *= 191) where a woman and a male adult family member were interviewed. Households where a woman was empowered irrespective of the adult male's score, or where she was not empowered but her score was equal to or greater than her male pair's score, were classified as achieving gender parity; households where a woman was not empowered and her score was lower than the male pair's score were classified as households lacking gender parity.

11 Included persons empowered in the pro-WEAI indicators selected for study.

### Empowerment of participants

Women FBO members were more empowered than non-FBO members in overall empowerment and as measured in 5 of the 11 individual indicators (Table 1). The mean empowerment score for FBO members was higher than that of their counterparts (0.82 ± 0.13 compared with 0.73 ± 0.16; *P* < 0.001). The FBO women compared with non-FBO women reported a higher number of groups in which they were active members (1.65 ± 0.9 compared with 0.96 ± 0.7; *P* < 0.001) or influenced their community (1.31 ± 1.1 compared with 0.62 ± 0.7; *P* < 0.001) as well as access to services sources (1.09 ± 1.3 compared with 0.76 ± 0.94; *P *< 0.01).

The male pairs of FBO members had a higher empowerment score than male pairs of non-FBO members (0.83 ± 0.13 compared with 0.79 ± 0.14; *P* = 0.03). Similarly to the women, they reported a higher number of groups in which they were active members (1.42 ± 1.1 compared with 0.92 ± 0.86; *P*  < 0.001) or influenced their community (1.22 ± 1.2 compared with 0.77 ± 0.9 ; *P* < 0.01), and access to services sources (1.23 ± 1.3 compared with 0.83 ± 0.9; *P* < 0.02). Households of FBO members were more likely to achieve gender parity (Table 1).

Women without a male pair were more likely to be empowered in household productive decisions (93.3% compared with 86.2%; *P* < 0.05), ownership of land and other assets (90.9% compared with 81.5%; *P* < 0.05), and control over the use of income (90.1% compared with 81.0%; *P* < 0.05). Compared with the first phase, being part of the second phase of the project was associated with women being more empowered (48.5% compared with 61.2%; *P* < 0.05), having a higher empowerment score (0.75 ± 0.2 compared with 0.79 ± 0.1; *P* < 0.01), and being more empowered in attitudes about domestic violence (66.1% compared with 76.4%; *P* < 0.05), access to and decisions on financial services (57.6% compared with 68.5%; *P* < 0.05), and membership in influential groups (53.9% compared with 69.7%; *P* < 0.01). In contrast, men in the first phase were more empowered (74.6% compared with 36.7%; *P* < 0.02), had a higher empowerment score (0.84 ± 0.1 compared with 0.79 ± 0.1; *P* < 0.01), and were more empowered in mobility (80.5% compared with 54.0%; *P* < 0.001), group membership (81.4% compared with 62.1%; *P* < 0.01), and membership in influential groups (70.3% compared with 47.1%; *P* < 0.01) than those in the second phase. Household gender parity did not differ between the 2 project phases.

### Primary analysis

#### Empowerment and women's FBO membership

In the adjusted model for all women, the odds of being empowered were 3.3 times higher for FBO members than for nonmembers (Table 2). The results were similar when the models were run separately for women with an adult male pair [adjusted odds ratio (aOR): 3.22; 95% CI: 1.67, 6.19] and those without a pair (aOR: 2.96; 95% CI: 1.23, 7.09). On the other hand, women's FBO membership was not associated with empowerment of the male family member. Households of women participating in FBOs were 2.8 times more likely to achieve gender parity. Secondary or higher education increased the odds of women's empowerment by >2-fold and household gender parity by ∼4-fold.

**TABLE 2:** Association of women's FBO participation with women's and men's empowerment and household gender parity in rural Ghana^1^

	Women's empowerment^2^ (*n *= 316)	Men's empowerment^3^ (*n *= 198)	Household gender parity^4^ (*n *= 191)
Women's FBO membership^5^ (ref.: not member)			
Member	3.25 (1.97, 5.33)***	1.53 (0.80, 2.92)	2.82 (1.39, 5.84)**
Individual			
Women's age group, y (ref.: <35)			
35–44	2.09 (0.90, 4.87)	—	2.07 (0.73, 9.96)
45–54	2.43 (0.97, 6.09)	—	1.98 (0.39, 9.94)
≥55	1.03 (0.40, 2.64)	—	0.87 (0.15, 5.10)
Men's age group, y (ref.: <35)			
35–44	—	0.61 (0.18, 2.10)	1.73 (0.43, 6.89)
45–54	—	0.63 (0.18, 2.16)	1.75 (0.35, 8.68)
≥55	—	1.36 (0.38, 4.83)	1.60 (0.27, 9.36)
Women's education^6^ (ref.: none)			
Primary	1.43 (0.70, 2.89)	—	1.22 (0.46, 3.21)
Secondary or higher	2.64 (1.22, 5.68)**	—	4.00 (1.40, 11.46)**
Men's education^6^ (ref.: none)			
Primary	—	1.17 (0.31, 4.34)	0.55 (0.11, 2.63)
Secondary or higher	—	1.96 (0.58, 6.67)	0.50 (0.11, 2.13)
Marital status^7^ (ref.: not married or cohabiting)			
Married/cohabiting	0.69 (0.37, 1.30)	—	—
Household			
Size, *n*	0.84 (0.74, 0.96)*	0.87 (0.75, 1.02)	0.91 (0.76, 1.08)
Phase of enrollment (ref.: phase 1)			
Phase 2	1.54 (0.93, 2.54)	0.50 (0.25, 0.97)*	1.91 (0.95, 3.85)^†^
Intercept	0.69 (0.17, 2.75)**	3.42 (0.52, 22.52)*	0.70 (0.07, 6.90)*

1 Values are ORs (95% CIs adjusted for multiple group comparisons using Dunnett's method) from generalized linear mixed models that were adjusted for the random effect of clusters. Empowerment outcomes measured using the project-level Women's Empowerment in Agriculture Index (35). Empowered: scored ≥80% in the 11 empowerment indicators (≥0.80). Household gender parity was calculated only for the households (*n *= 191) where a woman and a male adult family member were interviewed. Households where a woman was empowered irrespective of the adult male's score, or where she was not empowered but her score was equal to or greater than her male pair's score, were classified as achieving gender parity; households where a woman was not empowered and her score was lower than the male pair's score were classified as households lacking gender parity. FBO, farmer-based organization.

2 Model included all women participants from both paired (male and female) and female-only households with complete data for all variables.

3 Model included all men with complete data for all variables .

4 Model includes households with complete data for all variables for both the woman and the male adult family member (*n *= 191).

5 Woman in the household was participating in an FBO.

6 Highest level of education completed.

7 Married/cohabiting compared with not married or cohabiting.

^†^ *P *< 0.1, *P < 0.05, **P < 0.01, ***P < 0.001.

#### FBO membership and empowerment with nutrition outcomes

Women's FBO membership and empowerment were not associated with women's and men's BMI (Table 3). In all adjusted models, the likelihood of household food insecurity was higher among households where a woman was participating in FBOs (Table 3). Overall women's empowerment was not associated with household food insecurity in both models including all women and women from households with a male family member. Among paired households, male empowerment was negatively associated with household food insecurity [adjusted β coefficient (aβ): −0.79; 95% CI: −1.58, −0.01] (Table 3). Household gender parity was not associated with household food insecurity.

**TABLE 3:** Association of women's and men's nutritional status and household food security with women's participation in FBOs, women's and men's empowerment, and household gender parity in rural Ghana^1^

	Women's BMI^2^			Men's BMI^2^			Household food security^3^		
	Model 1^4^	Model 2^5^	Model 3^5^	Model 1^4^	Model 2^5^	Model 3^5^	Model 1^4^	Model 2^5^	Model 3^5^
Women's FBO membership^6^ (ref.: not member)									
Member	0.91 (−0.31, 2.35)	0.22 (−1.80, 2.24)	0.25 (−1.74, 2.25)	−0.03 (−2.76, 2.70)	−0.02 (−2.77, 2.71)	−0.09 (−2.80, 2.60)	0.53 (0.02, 1.03)*	0.78 (0.08, 1.48)*	0.73 (0.05, 1.42)*
Women's empowerment^7^ (ref.: not empowered)									
Empowered	0.80 (−0.65, 2.27)	1.10 (−0.99, 3.20)	—	0.45 (−2.24, 3.16)	0.24 (−2.59, 3.07)	—	−0.41 (−0.93, 0.10)	−0.42 (−1.16, 0.31)	—
Men's empowerment^7^ (ref.: not empowered)									
Empowered	—	−1.22 (−3.43, 0.98)	—	—	0.78 (−2.21, 3.78)	—	—	−0.79 (−1.58, −0.01)*	—
Household gender parity^7^ (ref.: no)									
Yes	—	—	0.89 (−1.28, 3.07)	—	—	0.93 (−1.99, 3.86)	—	—	−0.48 (−1.24, 0.27)
Individual									
Women's age group, y (ref.: <35)									
35–44	0.96 (−1.39, 3.33)	2.72 (−0.89, 6.35)	2.87 (−0.74, 6.48)	−1.75 (−6.58, 3.08)	−1.60 (−6.49, 3.28)	−1.87 (−6.73, 2.98)	0.75 (−0.07, 1.57)	0.92 (−0.34, 2.18)	0.96 (−0.27, 2.20)
45–54	−0.48 (−3.00, 2.02)	0.71 (−3.82, 5.26)	0.92 (−3.60, 5.45)	2.67 (−3.42, 8.77)	2.80 (−3.33, 8.94)	2.63 (−3.45, 8.72)	0.65 (−0.21, 1.52)	0.82 (−0.76, 2.41)	0.76 (−0.81, 2.34)
≥55	−1.21 (−3.76, 1.33)	0.82 (−4.35, 5.99)	0.97 (−4.19, 6.14)	−1.56 (−8.52, 5.38)	−1.46 (−8.44, 5.50)	−1.56 (−8.50, 5.38)	0.43 (−0.44, 1.30)	1.19 (−0.63, 3.02)	1.37 (−0.43, 3.19)
Men's age group, y (ref.: <35)									
35–44	—	−0.85 (−4.90, 3.19)	−0.90 (−4.95, 3.15)	2.20 (−3.24, 7.65)	2.22 (−3.23, 7.68)	2.12 (−3.33, 7.57)	—	0.63 (−0.73, 2.01)	0.67 (−0.69, 2.04)
45–54	—	0.00 (−4.60, 4.61)	0.01 (−4.68, 4.53)	1.18 (−5.04, 7.41)	1.16 (−5.08, 7.41)	1.11 (−5.11, 7.34)	—	−0.20 (−1.82, 1.40)	−0.17 (−1.77, 1.41)
≥55	—	−0.81 (−6.01, 4.38)	−1.08 (−6.25, 4.09)	3.36 (−3.60, 10.32)	3.21 (−3.79, 10.22)	3.30 (−3.65, 10.27)	—	−0.39 (−2.20, 1.40)	−0.50 (−2.29, 1.28)
Women's education^8^ (ref.: none)									
Primary	0.71 (−1.31, 2.73)	0.87 (−2.01, 3.76)	0.84 (−2.04, 3.73)	0.43 (−3.45, 4.33)	0.41 (−3.48, 4.31)	0.41 (−3.47, 4.30)	−0.13 (−0.83, 0.56)	0.18 (−0.79, 1.16)	0.26 (−0.71, 1.24)
Secondary or higher	−0.01 (−2.22, 2.20)	−0.40 (−3.36, 2.55)	−0.38 (−3.35, 2.58)	2.01 (−1.99, 6.03)	2.07 (−1.96, 6.10)	1.89 (−2.14, 5.92)	−0.05 (−0.80, 0.69)	0.45 (−0.55, 1.46)	0.45 (−0.54, 1.45)
Men's education^8^ (ref.: none)									
Primary	—	2.55 (−1.79, 6.91)	2.53 (−1.82, 6.88)	−1.44 (−7.30, 4.41)	−1.50 (−7.37, 4.37)	−1.35 (−7.21, 4.51)	—	1.08 (−0.43, 2.60)	0.92 (−0.57, 2.43)
Secondary or higher	—	1.99 (−2.03, 6.01)	1.88 (−2.13, 5.90)	1.01 (−4.38, 6.40)	0.89 (−4.53, 6.32)	1.12 (−4.28, 6.53)	—	0.76 (−0.63, 2.17)	0.52 (−0.86, 1.91)
Household									
Size, *n*	—	—	—	—	—	—	—	—	0.17 (−0.01, 0.36)^†^
Wealth^9^ (ref.: low)									
Medium	−1.27 (−3.22, 0.67)	−0.26 (−3.03, 2.51)	−0.28 (−3.06, 2.48)	−1.48 (−5.22, 2.25)	−1.45 (−5.20, 2.29)	−1.54 (−5.29, 2.19)	—	—	—
High	1.10 (−0.92, 3.13)	1.74 (−0.99, 4.49)	1.70 (−1.03, 4.44)	−0.26 (−4.01, 3.47)	−0.29 (−4.04, 3.45)	−0.26 (−4.00, 3.47)	—	—	—
Phase of enrollment (ref.: phase 1)									
Phase 2	0.20 (−1.39, 1.80)	−0.37 (−2.35, 1.60)	−0.25 (−2.21, 1.70)	3.73 (0.81, 6.64)*	3.88 (0.90, 6.86)*	3.63 (0.71, 6.56)*	−0.44 (−0.95, 0.06)^†^	−0.44 (−1.15, 0.26)	−0.31 (−1.02, 0.38)
Intercept	24.58 (21.29, 27.88)***	22.61 (17.29, 27.92)***	21.78 (16.57, 26.98)***	22.52 (15.01, 30.03)***	22.14 (14.49, 29.79)***	22.29 (14.73, 29.84)***	0.29 (−0.75, 1.34)^†^	−0.68 (−2.97, 1.61)	−1.19 (−4.51, 0.52)

1 Values shown are β coefficients (95% CIs adjusted for multiple group comparisons using Dunnett's method) from generalized linear mixed models that were adjusted for the random effect of clusters. Each column represents a single mixed-effects model adjusted for covariates with the outcome variables. FBO, farmer-based organization.

2 BMI was calculated as weight (kg)/height (m^2^).

3 Food security was classified based on the 15-item Food Insecurity Experience Scale (36), as food secure and food insecure (which included mildly, moderately, and severely food insecure).

4 Model included all women participants from both paired (male and female) and female-only households with complete data for all variables (*n *= 316).

5 Model included only households with complete data for all variables for both the woman and the male adult family member (*n *= 191).

6 Woman in the household was participating in an FBO.

7 Empowerment outcomes were measured using the project-level Women's Empowerment in Agriculture Index (35). Empowered: scored ≥80% in the 11 empowerment indicators (≥0.80). Household gender parity was calculated only for the households (*n *= 191) where a woman and a male adult family member were interviewed. Households where a woman was empowered irrespective of the adult male's score, or where she was not empowered but her score was equal to or greater than her male pair's score, were classified as achieving gender parity; households where a woman was not empowered and her score was lower than the male pair's score were classified as households lacking gender parity.

8 Highest level of education completed.

9 Wealth was categorized by tertile for the first component of a principal components analysis of 18 household assets [improved water source, floor materials, wall materials, roof materials, toilet facility, cooking fuel, ownership of agricultural land, small livestock, nonmechanized farm equipment (i.e., hand tools), mechanized farm equipment (i.e., tractor), house or building, electricity, motorcycle, bicycle, cellphone, radio, television, and refrigerator].

^†^ *P *< 0.1, *P < 0.05, ***P < 0.001.

### Secondary analysis

#### FBO membership and individual empowerment indicators

Women's FBO membership was positively associated with the individual indicators of women's empowerment related to attitudes about domestic violence (aOR: 1.66; 95% CI: 0.99, 2.76), access to and decisions on financial services (aOR: 1.71; 95% CI: 1.05, 2.76), mobility (aOR: 1.98; 95% CI: 1.18, 3.32), group membership (aOR: 2.74; 95% CI: 1.42, 5.26), and membership in influential groups (aOR: 3.12; 95% CI: 1.87, 5.21) (**Supplemental Table 1**). Women's FBO participation was not associated with men's individual empowerment indicators (**Supplemental Table 2**). Our ex post power analysis showed we were powered to detect differences in the empowerment indicators for women's models but not men's models (**Supplemental Table 3**).

#### Individual empowerment indicators with nutrition indicators

There was no significant association of the 5 indicators of individual empowerment with women's and men's BMI (**Supplemental Table 4**). Women's empowerment related to attitudes about domestic violence was negatively associated with household food insecurity (aβ: −0.78; 95% CI: −1.35, 0.21). Whereas, empowerment in access to and decisions on financial services was positively associated with household food insecurity among women (aβ: 0.88; 95% CI: 0.35, 1.14) and men (aβ: 0.97; 95% CI: 0.17, 1.77) (Supplemental Tables 4 and **5**).

## Discussion

Our analysis demonstrated that women's FBO membership was associated with a greater likelihood of their overall empowerment, and with specific indicators of attitudes about domestic violence, access to and decisions on financial services, mobility, group membership, and membership in influential groups. Our findings are consistent with studies that show group participation contributes to women's empowerment (10, 21). Brody et al. (21) included qualitative studies in a systematic review and provided insight about pathways to empowerment through self-help group (SHG) participation. Female members reported improvements in their self-confidence, and they were more confident speaking in public. The enhanced respect from husbands, other household members, and community members made a way for women to participate more in household decisions. The decrease in experiences of domestic violence among members was attributed to solidarity within the groups. Women's participation equipped them with financial skills, which is not surprising given that services and savings activities are often core to SHG activities. Finally, the SHGs made women more aware of their rights through involvement in social activities, built their social networks, and enabled them to take on leadership roles within their communities. Indeed, in our study there was evidence of leadership characteristics among FBO members. In comparison with non-FBO members, they were more likely to be active members of other groups and participate in groups that had an influence within their communities. This may reflect a difference in the leadership capabilities of women who join an FBO as well as suggest that FBOs may promote members to join and be active in other groups. Group-based approaches that facilitate programs to improve the empowerment of rural women can be expected to enhance the well-being of women and their families (22, 41, 42). Brody et al. (21) found that participation in SHGs improved women's economic and political empowerment, mobility, and decisions regarding their reproductive health (effect sizes ranging from 0.06 to 0.41 SD).

In the present study, we found that the likelihood of household gender equality was higher in households where a woman was participating in an FBO. However, women's FBO membership was not associated with overall male empowerment. Similarly, in India, women's SHG membership was associated with lower household inequality, with a 34% reduction in the difference between women's and men's empowerment scores (23). However, in contrast to our results, women's SHG participation was associated with men's empowerment in the domains of decisions on financial services and control over income. The lack of a relation between women's FBO participation and male empowerment in our analysis does not suggest the absence of a relation because our ex post power analysis showed we were underpowered to detect a difference if it existed among our male sample. We did not have a large enough sample of male participants given that 25% of our households were female-only and 12% of the households did not have a man available for interview at the time of the surveys. We recommend that future studies take this into consideration when calculating their sample size. The finding that women's participation in FBOs may contribute to reducing the gender gap in empowerment has important implications for rural women and the Ghana agriculture sector. Closing the gender gap in agriculture in low-resource countries could result in a 2.5%–4% increase in agricultural output, hence contributing to food security (43).

Women's group membership does not appear to affect all areas of empowerment. Kumar et al. (23) found in their study in India that SHG membership was only weakly associated with women's ownership of assets. In Uganda, a study reported that women's membership in agriculture cooperatives did not change the domestic and farm-related division of labor for the household (44). There may be different reasons why group membership may not affect all indicators of empowerment. The groups may vary in their characteristics, such as the type (mixed compared with women only; functional activities), sociocultural norms, and involvement of men in group activities that promote changes in gender roles and expectations (10, 12, 45). These factors can constitute barriers to women's active participation within the farmer organizations (46). In addition, groups may be more focused toward improving women's incomes and community development rather than challenging social norms embedded within societies that disempower women (21). For empowerment to occur, women have to be active agents (9, 47). There is a need to further integrate gender-sensitive strategies within farmer groups to promote women's active participation and empowerment.

In the current study, households of women participating in FBOs were more likely to be food insecure and had a higher BMI than nonmembers, suggesting that group membership alone may not be sufficient to improve nutrition-related indicators. In a review of South Asia studies, authors reported that group-based approaches that lacked clear nutrition goals and strategies were less likely to achieve nutrition impact (48). Integrating transformative approaches like nutrition behavior change communication together with gender sensitization in groups may be important to maximize the nutrition benefits of FBOs among rural women farmers in Ghana (22, 48).

Kumar et al. (48) proposed 4 pathways to nutrition impact through women's group–based approaches with women's empowerment highlighted as 1 of the essential components for achieving impact. There is evidence showing that different dimensions of empowerment affect individual and household nutrition (22, 42, 49, 50). Moreover, many of the dimensions associated with nutrition are extrinsic in nature and may be influenced by active group participation (42). For instance, in Ghana, women's empowerment in the domains of income and production was positively associated with household availability of macronutrients; women's control over income was the highest predictor of nutrient intake (51). Women's land ownership has been linked also with higher budget shares allocated to food in the household (17). In the present study, overall women's empowerment was not associated with adult nutritional status and household food security. Consistent with our findings, Quisumbing et al. (15) found that overall women's empowerment was not associated with women's BMI, women's dietary diversity score, as well as household dietary diversity score, particularly among the analyses conducted with data from African countries. In this study, male overall empowerment was a better predictor of household food security. In a meta-analysis, households headed by men were found to be less food insecure than female-headed households (52). The focus of recent interventions on women only appears contrary to these results. Households where both women and men jointly received information on market access and nutrition compared with women only have shown better food security indicators (53). This further highlights the need to include men in nutrition-sensitive agriculture interventions aiming to empower women and improve the nutrition of the household.

The individual empowerment indicators showed different associations with household food security than overall empowerment. For example, empowerment in access to and decisions on financial services for both women and men was associated with a higher likelihood of household food insecurity. This was unexpected given that studies have shown that household access to credit and women's decision making over credit were positively linked with household food security (19, 50, 54). Our finding perhaps reflects the strain in some households of borrowing at high interest rates. On the other hand, women's empowerment related to attitudes about domestic violence was associated with a decreased likelihood of household food insecurity. Among married women in Nepal, food insecurity has been associated with a higher likelihood of intimate partner violence (55). Local policies and nutrition-sensitive agriculture interventions focused on improving household food security and women's empowerment within the studied context could focus on addressing these 2 indicators.

Our assessment of the relation between women's FBO membership and empowerment has limitations. First, women voluntarily joined and participated in the FBOs in their communities before the study. Although we did not find any significant difference between FBO and non-FBO members in terms of demographic characteristics, women who join FBOs may be different across unobservable characteristics related to the different domains, introducing selection bias into our estimates. Second, our study design limited our ability to infer the direction of causality. Women who joined groups and their male counterparts may have been empowered before joining the group or increased their empowerment through participation before this study. Although we acknowledge the first 2 limitations of the study, the pro-WEAI tool has intrinsic and extrinsic indicators that allow one to make a case for the finding that FBOs may influence women's empowerment. The indicators with associations with FBO membership for women, with the exception of attitudes about domestic violence, were less intrinsic, meaning they are likely to be influenced by activities related to the group, which may then suggest some contribution of the FBOs. Finally, we had a combination of mixed and women-only groups, and we did not assess women's participation level in the FBOs. Although the selected FBOs had high female membership and were active within their community, individual variation in the level of participation existed. The influence that highly active participation may have for both women's and men's empowerment may be underestimated. The results should be interpreted with caution against these limitations.

Despite the limitations, the study contributes to the few studies that have examined the role of women's participation in farmer groups on women's and men's empowerment, as well as the linkage between empowerment in agriculture and nutrition-related indicators in the African context. The results suggest that FBOs in Ghana are an important tool to promote empowerment in nutrition-sensitive agriculture interventions for rural communities, although in combination with nutrition education, other gender-sensitive measures, and a better understanding of the impact on the different dimensions of empowerment.

Finally, our results show the outcomes of women's participation within existing groups designed to promote Ghana's agriculture and rural development policy. Women's participation within these groups needs to be well understood so effective approaches can be implemented to maximize benefits, promote gender equality, and improve food security and nutrition.

## Supplementary Material

nzac121_Supplemental_File

## Data Availability

Data described in the article, code book, and analytic code will be made available (in deidentified form) upon request pending application to the principal investigator (GSM) and approval.

## Notes

Supported by the Canadian Queen Elizabeth II Diamond Jubilee Scholarships (QES) (to AA). The QES is managed through a unique partnership of Universities Canada, Rideau Hall Foundation (RHF), Community Foundations of Canada (CFC), and Canadian universities. The Queen Elizabeth Scholarship-Advanced Scholars (QES-AS) is made possible with financial support from the International Development Research Center (IDRC) and Social Sciences and Humanities Research Council (SSHRC). The declared funders had no role in the design of the study; the collection, management, analysis, and interpretation of the data; as well as the preparation, review, and approval of the manuscript.

Author disclosures: the authors report no conflicts of interest.

GSM is an Associate Editor on *Advances in Nutrition* and the series’ Deputy Editor on Current Developments in Nutrition and played no role in the Journal's evaluation of the manuscript.

Supplemental Tables 1–5 are available from the “Supplementary data” link in the online posting of the article and from the same link in the online table of contents at https://academic.oup.com/cdn/.

Abbreviations used: AEA, agriculture extension agent; aOR, adjusted odds ratio; aβ, adjusted β coefficient; FBO, farmer-based organization; LINkINg Up, Scaling Up Women's Agripreneurship through Public-Private Linkages to Improve Rural Women's Income, Nutrition, and the Effectiveness of Institutions in Rural Ghana; LMKD, Lower Manya Krobo District; NGO, nongovernmental organization; NL, Nutrition Links; pro-WEAI, Project-level Women's Empowerment in Agriculture Index; SHG, self-help group; UMKD, Upper Manya Krobo District; YLKD, Yilo Krobo District.
