# Supplementary material for: The Association of Women's Participation in Farmer-Based Organizations with Female and Male Empowerment and its Implication for Nutrition-Sensitive Agriculture Interventions in Rural Ghana
Source: Curr Dev Nutr. 2022 Jul 25;6(9):nzac121. doi: 10.1093/cdn/nzac121 (PMC9469887; doi:10.1093/cdn/nzac121)
Supplement: nzac121_Supplemental_File [file nzac121_supplemental_file.docx]

**The association of women’s participation in farmer-based organizations with female and male empowerment and its implication for nutrition-sensitive agriculture interventions in rural Ghana**

Aishat Abdu

Online Supplementary Material

**SUPPLEMENTARY TABLE 1:** Association between women’s FBO participation with individual empowerment indicators^1^ among women in rural Ghana

|  | Attitudes about domestic violence | Access and decisions on financial services | Mobility | Group  membership | Membership  in influential groups |
| --- | --- | --- | --- | --- | --- |
| Women’s FBO membership^2^ |  |  |  |  |  |
| Member | 1.66 (0.99, 2.76) ^*^ | 1.71 (1.05, 2.76) ^*^ | 1.98 (1.18, 3.32) ^**^ | 2.74 (1.42, 5.26) ^**^ | 3.12 (1.87, 5.21) ^***^ |
| Not member *(ref)* |  |  |  |  |  |
| **Individual** |  |  |  |  |  |
| Women’s age group, y |  |  |  |  |  |
| 35-44 | 1.02 (0.42, 2.48) | 1.16 (0.51, 2.63) | 2.76 (1.14, 6.69) ^*^ | 1.12 (0.36, 3.45) | 0.92 (0.38, 2.22) |
| 45-54 | 0.84 (0.33, 2.12) | 1.31 (0.54, 3.19) | 2.37 (0.93, 6.02) | 1.08 (0.33, 3.49) | 0.98 (0.38, 2.49) |
| ≥ 55 | 0.82 (0.31, 2.16) | 1.01 (0.40, 2.52) | 1.38 (0.53, 3.54) | 0.53 (0.16, 1.68) | 0.66 (0.25, 1.74) |
| < 35 *(ref)* |  |  |  |  |  |
| Women’s education^3^ |  |  |  |  |  |
| Primary | 1.27 (0.62, 2.60) | 1.30 (0.66, 2.58) | 0.76 (0.36, 1.61) | 1.26 (0.53, 2.97) | 0.86 (0.43, 1.73) |
| Secondary or higher | 1.41 (0.66, 3.03) | 1.78 (0.85, 3.72) | 0.93 (0.42, 2.07) | 1.79 (0.69, 4.65) | 2.64 (1.20, 5.79) ^*^ |
| None *(ref)* |  |  |  |  |  |
| Women’s marital status |  |  |  |  |  |
| Married/cohabiting | 1.55 (0.84, 2.85) | 0.76 (0.41, 1.40) | 1.63 (0.88, 3.02) | 1.10 (0.59, 2.03) | 1.17 (0.63, 2.18) |
| Not married/cohabiting *(ref)* |  |  |  |  |  |
| **Household** |  |  |  |  |  |
| Size, # | 0.87 (0.76, 0.99) ^*^ | 1.04 (0.92, 1.18) | 0.88 (0.77, 1.01) | 0.87 (0.74, 1.01) ^†^ | 0.93 (0.82, 1.06) |
| Phase of enrollment |  |  |  |  |  |
| Phase 2 | 1.83 (1.10, 3.05) | 1.57 (0.96, 2.58) | 0.76 (0.45, 1.29) | 1.10 (0.59, 2.03) | 1.69 (1.01, 2.82) ^*^ |
| Phase 1 *(ref)* |  |  |  |  |  |
| Intercept | 1.82 (2.12, 7.06) ^*^ | 1.47 (0.17, 2.61) | 1.67 (0.41, 6.71) ^**^ | 5.63 (1.09, 29.12) |  |

^†^ p<0.1, ^*^ p<0.05, ^**^ p<0.01, ^***^ p<0. 001.

Values shown are odd ratios (95% Confidence Intervals adjusted for multiple group comparisons using Dunnett’s method) from generalized linear mixed models that were adjusted for the random effect of clusters. All models included women participants from both paired (male and female) and female only households with complete data for all variables (n=316). FBO: farmer-based organization. ^1^Empowerment indicators measured using the project-level Women’s Empowerment in Agriculture Index (pro-WEAI) (35). Individual empowerment indicators were classified as adequate, based on the pre-determined thresholds for the pro-WEAI. P-values for the five different models that estimated the association between women’s FBO membership and empowerment were corrected for multiple hypothesis testing following the Benjamin *et al.* (2006) method for q-value corrections(39, 40). ^2^Woman in the household is participating in an FBO. ^3^Highest level of education completed.

**SUPPLEMENTARY TABLE 2:** Association between women’s FBO participation with individual empowerment indicators^1^ among men in rural Ghana

|  | Attitudes about domestic violence | Access and decisions on financial services | Mobility | Group  membership | Membership  in influential groups |
| --- | --- | --- | --- | --- | --- |
| Women’s FBO membership^2^ |  |  |  |  |  |
| Member | 1.58 (0.65, 3.83) | 2.14 (1.07, 4.28) | 0.80 (0.39, 1.64) | 2.11 (1.00, 4.46) | 1.76 (0.89, 3.47) |
| Not member *(ref)* |  |  |  |  |  |
| **Individual** |  |  |  |  |  |
| Women’s age group, y |  |  |  |  |  |
| 35-44 | 1.71 (0.35, 8.33) | 0.71 (0.19, 2.55) | 1.73 (0.46, 6.46) | 0.48 (0.12, 1.86) | 0.33 (0.09, 1.16) |
| 45-54 | 1.62 (0.22, 11.48) | 1.30 (0.26, 6.36) | 1.67 (0.30, 9.07) | 0.58 (0.11, 3.12) | 0.36 (0.07, 1.70) |
| ≥ 55 | 0.92 (0.09, 8.78) | 0.33 (0.05, 1.91) | 4.50 (0.55, 36.57) | 0.43 (0.06, 3.11) | 0.60 (0.10, 3.64) |
| < 35 *(ref)* |  |  |  |  |  |
| Men’s age group, y |  |  |  |  |  |
| 35-44 | 1.62 (0.29, 8.89) | 1.90 (0.46, 7.87) | 0.47 (0.10, 2.12) | 0.31 (0.25, 5.31) | 1.23 (0.30, 5.00) |
| 45-54 | 0.75 (0.11, 5.09) | 1.15 (0.24, 5.54) | 0.82 (0.14, 4.87) | 0.97 (0.17, 5.32) | 1.30 (0.26, 6.33) |
| ≥ 55 | 1.29 (0.14, 12.00) | 1.70 (0.29, 10.06) | 0.47 (0.06, 3.42) | 2.67 (0.38, 18.63) | 2.28 (0.40, 12.97) |
| < 35 *(ref)* |  |  |  |  |  |
| Women’s education^3^ |  |  |  |  |  |
| Primary | 1.38 (0.34, 5.57) | 0.95 (0.36, 2.50) | 0.74 (0.24, 2.25) | 0.92 (0.31, 2.73) | 0.90 (0.33, 2.42) |
| Secondary or higher | 0.64 (0.18, 2.28) | 2.27 (0.82, 6.25) | 0.61 (0.20, 1.86) | 1.15 (0.38, 3.52) | 0.96 (0.53, 2.63) |
| None *(ref)* |  |  |  |  |  |
| Men’s education^3^ |  |  |  |  |  |
| Primary | 1.35 (0.16, 10.87) | 1.32 (0.31, 5.65) | 0.10 (0.09, 2.63) | 1.62 (0.31, 8.31) | 1.83 (0.42, 7.89) |
| Secondary or higher | 0.80 (0.12, 5.23) | 1.47 (0.39, 5.57) | 0.19 (0.17, 3.95) | 1.46 (0.33, 6.39) | 3.57 (0.91, 14.02) ^†^ |
| None *(ref)* |  |  |  |  |  |
| **Household** |  |  |  |  |  |
| Size, # | 1.06 (0.84, 1.34) | 0.93 (0.79, 1.10) | 1.01 (0.85, 1.21) | 0.89 (0.75, 1.06) | 0.96 (0.81, 1.13) |
| Phase of enrollment |  |  |  |  |  |
| Phase 2 | 0.64 (0.21, 1.98) | 0.90 (0.46, 1.78) | 0.61 (0.23, 1.65) ^***^ | 1.15 (0.42, 3.12) ^**^ | 0.96 (0.39, 2.36) ^**^ |
| Phase 1 *(ref)* |  |  |  |  |  |
| Intercept | 2.30 (9.77, 51.88) ^†^ | 1,10 (0.12, 9.97) ^†^ | 8.88 (0.57, 137.52) | 4.81 (0.37, 62.60) ^*^ | 1.17 (0.10, 12.56) |

^†^ p<0.1, ^*^ p<0.05, ^**^ p<0.01, ^***^ p<0. 001.

Values shown are odd ratios (95% Confidence Intervals adjusted for multiple group comparisons using Dunnett’s method) from generalized linear mixed models that were adjusted for the random effect of clusters. All models included adult male family members from paired (male and female) households with complete data for all variables (n=191). FBO: farmer-based organization. ^1^Empowerment indicators measured using the project-level Women’s Empowerment in Agriculture Index (pro-WEAI) (35). Individual empowerment indicators were classified as adequate, based on the pre-determined thresholds for the pro-WEAI. P-values for the five different models that estimated the association between women’s FBO membership and empowerment were corrected for multiple hypothesis testing following the Benjamin *et al.* (2006) method for q-value corrections (39, 40). ^2^Woman in the household is participating in an FBO. ^3^Highest level of education completed.

**SUPPLEMENTARY TABLE 3.** Minimum detectable difference in empowerment indicators^1^ by women’s FBO membership^2^

|  | Empowerment | Attitudes about domestic violence | Access and decisions on financial services | Mobility | Group  membership | Membership  in influential groups |
| --- | --- | --- | --- | --- | --- | --- |
| Women | 0.16 | 0.14 | 0.15 | 0.15 | 0.12 | 0.16 |
| Men | 0.18 | 0.13 | 0.18 | 0.17 | 0.18 | 0.20 |

^1^Empowerment indicators measured using the project-level Women’s Empowerment in Agriculture Index (pro-WEAI) (35). Empowered: scored at least 80% or greater in the 11 empowerment indicators (≥0.80). Individual empowerment indicators were classified as adequate, based on the pre-determined thresholds for the pro-WEAI. ^2^Woman in the household is participating in an FBO.

**SUPPLEMENTARY TABLE 4:** Association between women’s and men’s nutritional status and household food security with individual empowerment indicators among women^1^ in rural Ghana

|  | Women’s BMI^2,4^  (n=316) | Men’s BMI^2,5^  (n=191) | Household food security^3,4^  (n=316) |
| --- | --- | --- | --- |
| Attitudes about domestic violence |  |  |  |
| Empowered | 0.97 (-0.59, 2.54) | 1.04 (-1.98, 4.06) | -0.78 (-1.35, -0.21) ^**^ |
| Not empowered *(ref)* |  |  |  |
| Access to and decisions on financial service |  |  |  |
| Empowered | 0.68 (-0.78, 2.15) | -0.79 (-3.48, 1.89) | 0.88 (0.35, 1.41) ^**^ |
| Not empowered *(ref)* |  |  |  |
| Mobility |  |  |  |
| Empowered | -0.73 (-2.32, 0.85) | 1.13 (-1.94, 4.20) | 0.11 (-0.44, 0.67) |
| Not empowered *(ref)* |  |  |  |
| Group membership |  |  |  |
| Empowered | 0.74 (-1.64, 3.12) | 0.69 (-3.98, 5.37) | 0.43 (-0.40, 1.27) |
| Not empowered *(ref)* |  |  |  |
| Membership in influential groups | 0.97 (-0.89, 2.83) | -0.85 (-4.48, 2.78) | -0.44 (-1.11, 0.22) |
| Empowered |  |  |  |
| Not empowered *(ref)* |  |  |  |
| **Individual** |  |  |  |
| Women’s age group, y |  |  |  |
| 35-44 | 1.23 (-1.14, 3.60) | -2.01 (-6.99, 2.95) | 0.69 (-0.15, 1.54) |
| 45-54 | -0.01 (-2.55, 2.52) | 1.94 (-4.39, 8.28) | 0.53 (-0.20, 1.27) |
| ≥ 55 | -0.75 (-3.32, 1.81) | -1.83 (-8.94, 5.28) | 0.36 (-0.38, 1.11) |
| < 35 *(ref)* |  |  |  |
| Men’s Age group, y |  |  |  |
| 35-44 | ⎯ | 2.20 (-3.50, 7.92) | ⎯ |
| 45-54 | ⎯ | 1.78 (-4.74, 8.31) | ⎯ |
| ≥ 55 | ⎯ | 3.46 (-3.69, 10.62) | ⎯ |
| < 35 *(ref)* |  |  |  |
| Women’s education^6^ |  |  |  |
| Primary | 0.85 (-1.14, 2.86) | 0.68 (-3.29, 4.66) | -0.16 (-0.79, 0.47) |
| Secondary or higher | -0.08 (-2.26, 2.08) | 2.04 (-2.04, 6.13) | -0.16 (-0.85, 0.51) |
| None *(ref)* |  |  |  |
| Men’s education^6^ |  |  |  |
| Primary | ⎯ | -1.27 (-7.39, 4.83) | ⎯ |
| Secondary or higher | ⎯ | 1.24 (-4.38, 6.86) | ⎯ |
| None *(ref)* |  |  |  |
| **Household** |  |  |  |
| Wealth^7^ |  |  |  |
| Medium | -1.29 (-3.38, 0.79) | -1.68 (-5.78, 2.42) | ⎯ |
| High | 1.05 (-1.35, 3.46) | -0.73 (-5.44, 3.97) | ⎯ |
| Low *(ref)* |  |  |  |
| Phase of enrollment |  |  |  |
| Phase 2 | -0.09 (-1.57, 1.39) | 2.91 (-0.02, 5.86) ^†^ | -0.45 (-0.97, 0.07) |
| Phase 1 *(ref)* |  |  |  |
| Intercept | 23.30 (20.11, 26.49) ^***^ | 18.03 (9.55, 26.50) ^***^ | 0.30 (-1.02, 1.63) |

^†^ p<0.1 ^*^ p<0.05, ^**^ p<0.01, ^***^ p<0. 001.

Values shown are beta coefficients (95% Confidence Intervals adjusted for multiple group comparisons using Dunnett’s method) from generalized linear mixed models that were adjusted for the random effect of clusters. All five empowerment indicators included in the models as covariates. Multicollinearity between explanatory were checked by the variance inflation factor (VIF). BMI: body mass index.^1^Empowerment indicators measured using the project-level Women’s Empowerment in Agriculture Index (pro-WEAI) (35). Individual empowerment indicators were classified as adequate, based on the pre-determined thresholds for the pro-WEAI. ^2^BMI was calculated as weight (kg)/height (m^2^). ^3^Food security: classification based on the 15-item Food Insecurity Experience Scale (36). Food secure and food insecure (included mildly, moderately, and severely food insecure). ^4^Model included all women participants from both paired (male and female) and female only households with complete data for all variables (n=316). ^5^Model included only households with complete data for all variables for both the woman and the male adult family member (n=191). ^6^Highest level of education completed. ^7^Wealth: tertiles for the first component of a principal components analysis of 18 household assets (improved water source, floor materials, wall materials, roof materials, toilet facility, cooking fuel, ownership of agricultural land, small livestock, non-mechanized farm equipment (i.e., hand tools), mechanized farm equipment (i.e., tractor), house or building, electricity, motorcycle, bicycle, cellphone, radio, television, and refrigerator).**SUPPLEMENTARY TABLE 5:** Association between women’s and men’s nutritional status and household food security with individual empowerment indicators among men^1^ in rural Ghana

|  | Women’s BMI^2^  (n=191) | Men’s BMI^2^  (n=191) | Household food security^3^  (n=191) |
| --- | --- | --- | --- |
| Attitudes about domestic violence |  |  |  |
| Empowered | -2.33 (-5.05, 0.37) ^†^ | 1.67 (-2.04, 5.39) | -0.03 (-0.98, 0.90) |
| Not empowered *(ref)* |  |  |  |
| Access and decisions on financial service |  |  |  |
| Empowered | -0.53 (-2.76, 1.68) | -1.73 (-4.73, 1.25) | 0.97 (0.17, 1.77) ^*^ |
| Not empowered *(ref)* |  |  |  |
| Mobility |  |  |  |
| Empowered | 0.32 (-1.93, 2.57) | 1.58 (-1.54, 4.71) | 0.57 (-0.26, 1.40) |
| Not empowered *(ref)* |  |  |  |
| Group membership |  |  |  |
| Empowered | 1.07 (-2.29, 4.43) | 3.05 (-1.52, 7.63) | -0.88 (-2.16, 0.40) |
| Not empowered *(ref)* |  |  |  |
| Membership in influential groups | 0.62 (-2.37, 3.62) | -0.48 (-4.58, 3.60) | -1.02 (-2.10, 0.06) ^†^ |
| Empowered |  |  |  |
| Not empowered *(ref)* |  |  |  |
| **Individual** |  |  |  |
| Women’s Age group, y |  |  |  |
| 35-44 | 3.57 (-0.05, 7.19) ^†^ | -1.75 (-6.65, 3.15) | 0.85 (-0.43, 2.14) |
| 45-54 | 1.59 (-2.93, 6.11) | 2.32 (-3.87, 8.52) | 0.70 (-0.92, 2.33) |
| ≥ 55 | 1.14 (-4.04, 6.34) | -2.12 (-9.19, 4.94) | 1.46 (-0.45, 3.38) |
| < 35 *(ref)* |  |  |  |
| Men’s Age group, y |  |  |  |
| 35-44 | -0.71 (-4.77, 3.34) | 2.20 (-3.39, 7.79) | 0.61 (-0.80, 2.08) |
| 45-54 | -0.16 (-4.76, 4.43) | 1.55 (-4.78, 7.88) | -0.28 (-1.94, 1.38) |
| ≥ 55 | -1.26 (-6.46, 3.92) | 3.02 (-3.98, 10.04) | -0.37 (-2.25, 1.50) |
| < 35 *(ref)* |  |  |  |
| Women’s education^4^ |  |  |  |
| Primary | 1.04 (-1.82, 3.91) | 0.56 (-3.32, 4.45) | 0.12 (-0.88, 1.13) |
| Secondary or higher | -0.26 (-3.19, 2.66) | 2.06 (-1.19, 6.05) | 0.24 (-0.80, 1.29) |
| None *(ref)* |  |  |  |
| Men’s education^4^ |  |  |  |
| Primary | 2.45 (-1.88, 6.79) | -0.70 (-6.70, 5.28) | 1.35 (-0.18, 2.89) |
| Secondary or higher | 1.52 (-2.51, 5.57) | 1.58 (-4.05, 7.22) | 0.97 (-0.45, 2.41) |
| None *(ref)* |  |  |  |
| **Household** |  |  |  |
| Wealth^5^ |  |  |  |
| Medium | 0.25 (-2.54, 3.05) | -0.97 (-5.02, 3.07) | ⎯ |
| High | 1.88 (-0.86, 4.63) | -0.08 (-4.70, 4.52) | ⎯ |
| Low *(ref)* |  |  |  |
| Phase of enrollment |  |  |  |
| Phase 2 | 0.34 (-1.72, 2.41) | 3.54 (0.46, 6.63) ^*^ | -0.62 (-1.41, 0.16) |
| Phase 1 *(ref)* |  |  |  |
| Intercept | 22.74 (16.69, 28.80) ^***^ | 14.91 (6.09, 23.72) ^***^ |  |

^†^ p<0.1 ^*^ p<0.05, ^**^ p<0.01, ^***^ p<0. 001.

Values shown are beta coefficients (95% Confidence Intervals adjusted for multiple group comparisons using Dunnett’s method) from generalized linear mixed models that were adjusted for the random effect of clusters. All models included adult male family members from paired (male and female) households with complete data for all variables (n=191). All five empowerment indicators included in the models as covariates. Multicollinearity between explanatory were checked by the variance inflation factor (VIF). BMI: body mass index.^1^Empowerment indicators measured using the project-level Women’s Empowerment in Agriculture Index (pro-WEAI) (35). Individual empowerment indicators were classified as adequate, based on the pre-determined thresholds for the pro-WEAI. ^2^BMI was calculated as weight (kg)/height (m^2^). ^3^Food security: classification based on the 15-item Food Insecurity Experience Scale (36). Food secure and food insecure (included mildly, moderately, and severely food insecure).^4^Highest level of education completed. ^5^Wealth: tertiles for the first component of a principal components analysis of 18 household assets (improved water source, floor materials, wall materials, roof materials, toilet facility, cooking fuel, ownership of agricultural land, small livestock, non-mechanized farm equipment (i.e., hand tools), mechanized farm equipment (i.e., tractor), house or building, electricity, motorcycle, bicycle, cellphone, radio, television, and refrigerator).
